# Supplementary material for: Mutations in PCYT2 disrupt etherlipid biosynthesis and cause a complex hereditary spastic paraplegia
Source: Brain. 2019 Oct 22;142(11):3382–97. doi: 10.1093/brain/awz291 (PMC6821184; doi:10.1093/brain/awz291)
Supplement: awz291_Supplementary_Data [file awz291_supplementary_data.zip › awz291-Suppl_data/Supplementary_Data4.zip!/Supplementary information 3 - fibroblast lipidomics.zip!/Fibroblast lipidomics/Controle vs Patient2/Statistical plots and data/DG species/Random forest.pdf]

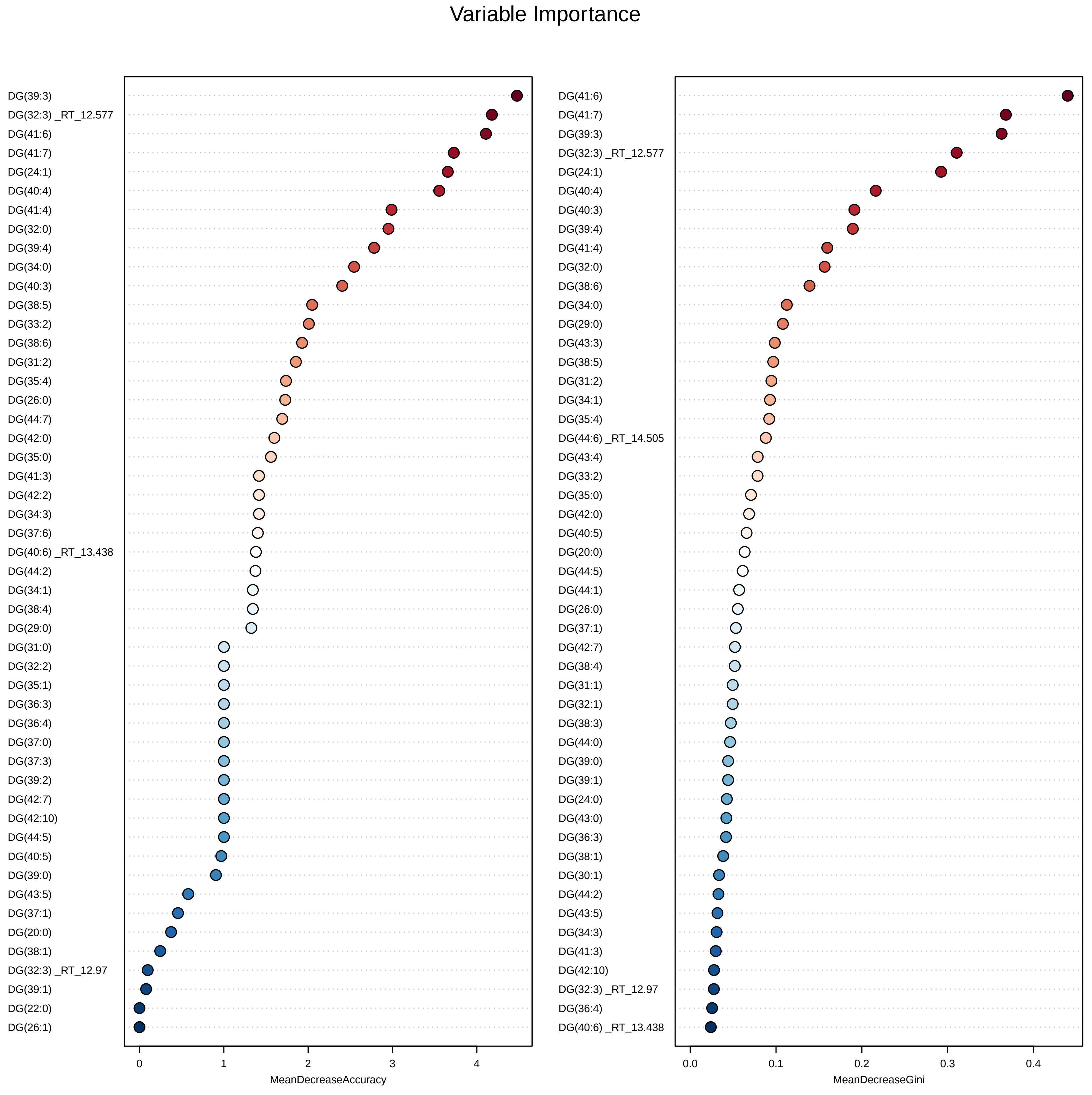
Variable Importance
DG(39:3)
DG(32:3) _RT_12.577
DG(41:6)
DG(41:7)
DG(24:1)
DG(40:4)
DG(41:4)
DG(32:0)
DG(39:4)
DG(34:0)
DG(40:3)
DG(38:5)
DG(33:2)
DG(38:6)
DG(31:2)
DG(35:4)
DG(26:0)
DG(44:7)
DG(42:0)
DG(35:0)
DG(41:3)
DG(42:2)
DG(34:3)
DG(37:6)
DG(40:6) _RT_13.438
DG(44:2)
DG(34:1)
DG(38:4)
DG(29:0)
DG(31:0)
DG(32:2)
DG(35:1)
DG(36:3)
DG(36:4)
DG(37:0)
DG(37:3)
DG(39:2)
DG(42:7)
DG(42:10)
DG(44:5)
DG(40:5)
DG(39:0)
DG(43:5)
DG(37:1)
DG(20:0)
DG(38:1)
DG(32:3) _RT_12.97
DG(39:1)
DG(22:0)
DG(26:1)
0
1
2
3
4
MeanDecreaseAccuracy
DG(41:6)
DG(41:7)
DG(39:3)
DG(32:3) _RT_12.577
DG(24:1)
DG(40:4)
DG(40:3)
DG(39:4)
DG(41:4)
DG(32:0)
DG(38:6)
DG(34:0)
DG(29:0)
DG(43:3)
DG(38:5)
DG(31:2)
DG(34:1)
DG(35:4)
DG(44:6) _RT_14.505
DG(43:4)
DG(33:2)
DG(35:0)
DG(42:0)
DG(40:5)
DG(20:0)
DG(44:5)
DG(44:1)
DG(26:0)
DG(37:1)
DG(42:7)
DG(38:4)
DG(31:1)
DG(32:1)
DG(38:3)
DG(44:0)
DG(39:0)
DG(39:1)
DG(24:0)
DG(43:0)
DG(36:3)
DG(38:1)
DG(30:1)
DG(44:2)
DG(43:5)
DG(34:3)
DG(41:3)
DG(42:10)
DG(32:3) _RT_12.97
DG(36:4)
DG(40:6) _RT_13.438
0.0
0.1
0.2
0.3
0.4
MeanDecreaseGini
